# Morgan County Medical Society

**Published:** 1868-07

**Authors:** C. J. Lucas

**Affiliations:** Sec’y pro tem.


### MORGAN COUNTY MEDICAL SOCIETY.

The regular monthly meeting of the Morgan County Medical Society was held in Music Hall, on Monday, April 8th, 1868, and was called to order by the President, Dr. Henry Jones, at 2 o'clock P.M.

In the absence of the Secretary, Dr. Lucas was elected Secretary *pro tem.*

The minutes of the last meeting were read and approved.

Drs. C. G. Jones and G. H. Sanford, both of Jacksonville, were nominated as members.

Dr. Prince exhibited some specimens of calculi from the bladder.

1. A specimen of four calculi, taken from a young man, 21 years old, by the lateral operation in 1857. They had been 15 years forming. The patient is still living.

2. A specimen of a single calculus of lithic acid, weighing 4¼ ounces, removed in 1859, by the lateral section. The patient is still living, though with an urinary fistula, which requires a plastic operation to close it.

3. A small phosphatic calculus, removed by the lateral section, from a boy 12 years old. The patient recovered, and is still living.

4. A nest of 14 calculi, of nearly the same shape and size, weighing, in the aggregate, 4 ounces (avoirdupois), taken from an old gentleman aged 63 years, in 1864. The patient recovered from the operation, and survived a year.

5. Three phosphatic calculi, weighing, in the aggregate, 5¼ ounces, removed from a patient 31 years of age, on the 5th of March last, by the median operation. The patient is in the process of recovery.

6. A phosphatic calculus, together with a small penknife, from a female bladder, removed by a section through the vaginal wall, which was subsequently closed by the operation usually performed for vesico-vaginal fistula. Recovery complete.

Comments were made upon the relations of these operations to general surgery, and upon questions which they helped to illustrate. One point made was, that after severe injuries and operations, a more supporting treatment is necessary, than accords with the extreme fear of inflammation, which prevailed in the first part of the present century.

Dr. Edgar thinks that if by diagnosis the calculus proves to be of large size, the operation of lateral incision is safer than that by dilatation and tearing, as the rectum is less likely to suffer. He made also some remarks about the formation of calculi in the bladder. He thinks that accidental detachment of mucus and foreign bodies is oftener the cause of their formation than certain qualities of food and water.

Dr. Bibb reported two cases of formation of calculus deposit, operations by Prof. Brainard, caused by the introduction of a button and part of a pencil, in the female bladder.

A letter from Dr. McVey was read, excusing himself for non-appearance. He promised to furnish the Society an essay some other time.

Dr. Lucas read an essay on the nature of lochia, and its abnormities in regard to color, quantity, quality, and odor, and reported a case of suppressed lochia of three days' standing, caused by a forcible contraction of the uterus, in connection with profuse sweating, and free suction of a strong child.

Dr. Prince made some remarks on the above subject. He compared the wound and its changes, produced by the detachment of the placenta from the uterus, with those of a surgical wound, and advised the study of surgery to every obstetrician.

Dr. Fisher asked the Society if bandaging after delivery was old fogyism, as considered by some physicians. He is of the opinion that the bandage is necessary for the expulsion of coagulated blood.

Dr. Edgar thinks it necessary in delicate persons, but not so important in stout ones. In connection he spoke about the diet and after-treatment of lying-in women, which ought to be nourishing and supporting. He also approves the knowledge of surgery to the obstetrician.

Dr. Reed remarked, the bandage is necessary to prevent the parts to remain loose and flabby, and to insure a good form, but gave a case where the woman was up the next day, without a bandage, doing her work. He referred also to the savages and half-civilized nations, women make little or no change in their general conduct in consequence of childbirth, but return to their usual occupation almost immediately after delivery.

Dr. Henry Jones stated, that in ascites, after throwing off the water, a bandage is applied to prevent a feeling of fainting and prostration; the same ought to be applied to the uterus after the expulsion of its contents, and sometimes on the above grounds before the expulsion of the afterbirth in tedious labors. He thinks that milk fever is a very rare occurrence and often only an imaginary disease.

Dr. Edgar is of the opinion that the febrile excitement, often called milk fever, is due to the reaction of the exhausting pain, depletion, and nervous depression. In regard to the application of the bandage, he thinks that the pressure is often not applied to its proper place, producing like tight-lacing, prolapse, tumors, displacement, and its consequences. Nine-tenths of the female troubles are, considered by him, due to faulty dress, and something ought to be done to prevent it.

Dr. Henry Jones thinks that all these remarks are of no use, for it is impracticable for a physician to correct tight-lacing.

Dr. Fisher is of the opinion that there is not so much imprudence in dress now as there used to be, and that the fashion controls what the physician cannot prevent.

Dr. Mitchell, of Meredosia, though not a member made some remarks on the propriety of the application of the bandage and the injury of imprudence of dress, corroborating the above opinions.

Drs. Edgar, Sr., Fisher, and Bibb were appointed a committee to make arrangements for the celebration of the Second Anniversary, to be held May 14th. On motion, the Society adjourned, to meet on the second Thursday of May, 1868, at 11 o'clock A.M., in the Fireman's Hall.

C. J. LUCAS, M.D., *Sec'y pro tem.*

